# The *Arabidopsis* Sin3-HDAC Complex Facilitates Temporal Histone Deacetylation at the *CCA1* and *PRR9* Loci for Robust Circadian Oscillation

**DOI:** 10.3389/fpls.2019.00171

**Published:** 2019-02-18

**Authors:** Hong Gil Lee, Cheljong Hong, Pil Joon Seo

**Affiliations:** ^1^Department of Chemistry, Seoul National University, Seoul, South Korea; ^2^Plant Genomics and Breeding Institute, Seoul National University, Seoul, South Korea

**Keywords:** circadian clock, chromatin modification, histone deacetylase (HDAC), Sin3 histone deacetylase and corepressor complex, CCA1, PRR9

## Abstract

The circadian clock synchronizes endogenous rhythmic processes with environmental cycles and maximizes plant fitness. Multiple regulatory layers shape circadian oscillation, and chromatin modification is emerging as an important scheme for precise circadian waveforms. Here, we report the role of an evolutionarily conserved Sin3-histone deacetylase complex (HDAC) in circadian oscillation in *Arabidopsis*. *SAP30 FUNCTION-RELATED 1* (*AFR1*) and *AFR2*, which are key components of Sin3-HDAC complex, are circadianly-regulated and possibly facilitate the temporal formation of the *Arabidopsis* Sin3-HDAC complex at dusk. The evening-expressed AFR proteins bind directly to the *CIRCADIAN CLOCK ASSOCIATED 1* (*CCA1*) and *PSEUDO-RESPONSE REGULATOR 9* (*PRR9*) promoters and catalyze histone 3 (H3) deacetylation at the cognate regions to repress expression, allowing the declining phase of their expression at dusk. In support, the *CCA1* and *PRR9* genes were de-repressed around dusk in the *afr1-1afr2-1* double mutant. These findings indicate that periodic histone deacetylation at the morning genes by the Sin3-HDAC complex contributes to robust circadian maintenance in higher plants.

## Introduction

The circadian clock is an internal time-keeper mechanism that ensures endogenous biological rhythms with a period of approximately 24 h, coinciding with daily environmental cycles. A large fraction of the plant transcriptome is clock-controlled, and thus the clock is globally linked to diverse signaling and metabolic pathways to ensure optimal biological functions at a specific time of day (Covington et al., 2008; Mizuno and Yamashino, 2008; Hsu and Harmer, 2012). Synchronization of the clock with the environment is closely associated with plant growth and fitness (Dodd et al., 2005; Fujiwara et al., 2008; Nusinow et al., 2011; Yoo et al., 2011; Lu et al., 2012; Nagel and Kay, 2012; Haydon et al., 2013; Zhang et al., 2013).

The circadian clock is a highly conserved system in higher eukaryotes. In *Arabidopsis*, the central oscillator is known to consist of an array of transcriptional loops. Two single-MYB transcription factors, CIRCADIAN CLOCK-ASSOCIATED 1 (CCA1) and LATE ELONGATED HYPOCOTYL (LHY), establish the central loop by repressing transcription of *TIMING OF CAB EXPRESSION 1* (*TOC1*) that in turn, represses *CCA1* and *LHY* expression (Alabadi et al., 2001; Huang et al., 2012; Pokhilko et al., 2013). The central loop is further regulated by PSEUDO-RESPONSE REGULATORs (PRR5, PRR7, and PRR9) (Nakamichi et al., 2005, 2010; Salome et al., 2010) and the evening complex (EC) consisting of EARLY FLOWERING 3 (ELF3), ELF4, and LUX ARRYTHMO/PHYTOCLOCK 1 (LUX/PCL1) (Nusinow et al., 2011; Chow et al., 2012; Herrero et al., 2012). Moreover, the TOC1 protein also plays widespread roles in transcriptionally repressing multiple core clock components, underscoring the biological importance of transcriptional regulation in circadian homeostasis (Gendron et al., 2012; Huang et al., 2012).

Accumulating evidence suggests that circadian oscillation is further shaped by additional regulatory mechanisms (Seo and Mas, 2014). In particular, chromatin modification is an important regulatory scheme underlying precise circadian waveforms (Mas, 2008; Stratmann and Mas, 2008; Kusakina and Dodd, 2012; Nagel and Kay, 2012). Transcript accumulation of core clock components correlates with rhythmic changes in accumulation of histone H3 acetylation (H3ac) in *Arabidopsis* (Hemmes et al., 2012; Malapeira et al., 2012; Song and Noh, 2012). Consistent with the fact that histone acetylation status is dynamically regulated by the antagonistic action of histone acetyltransferases (HATs) and histone deacetylases (HDACs) (Kuo and Allis, 1998; Yang and Seto, 2007), temporal association of specific sets of HATs and HDACs occurs at the loci of core clock components to shape rhythmic expression (Hemmes et al., 2012; Malapeira et al., 2012; Song and Noh, 2012). For instance, the midday-expressed HISTONE ACETYLTRANSFERASE OF THE TAFII250 FAMILY 2 (HAF2) protein catalyzes H3ac at the *PRR5* and *LUX* loci to activate expression and is responsible for the rising phase of *PRR5* and *LUX* circadian expression (Lee and Seo, 2018). In addition, the HDA6 and HDA19 proteins form protein complexes together with the TOPLESS (TPL) and PRR proteins, and repress expression of *CCA1* and *LHY* during the daytime (Wang et al., 2013). Despite the importance of diurnal histone acetylation states of core clock genes in stable circadian oscillation, the responsible epigenetic modifiers are yet to be fully characterized.

Histone deacetylase complex often form diverse types of multiprotein co-repressor complexes and play a variety of roles during plant growth and development (Buszewicz et al., 2016; Kim et al., 2016; Hung et al., 2018; Park et al., 2018; Tasset et al., 2018). One well-characterized HDAC complex in eukaryotes is the Sin3-HDAC complex (Alland et al., 2002; Kuzmichev et al., 2002; Silverstein and Ekwall, 2005; Clark et al., 2015). In *Arabidopsis*, the Sin3-HDAC complex participates in photoperiodic flowering through the periodic acetylation of the *FLOWERING LOCUS T* (*FT*) locus (Gu et al., 2013). The Sin3-HDAC complex is activated at the end of the day and is recruited to the *FT* locus by AGAMOUS LIKE 18 (AGL18) in a CONSTANS (CO)-dependent manner under long-day conditions (Gu et al., 2013). In this study, we report that the *Arabidopsis* Sin3-HDAC complex also temporally regulates *CCA1* and *PRR9* expression through catalyzing H3 deacetylation and facilitates the declining phase of their circadian expression during the evening time. These results reveal that temporal association of chromatin modifiers underlies robust rhythmic expression of clock genes and thereby stable circadian oscillation.

## Results

### Rhythmic Expression of *AFR*s Is Shaped by CCA1

Histone deacetylase complex often form multiprotein co-repressor complexes, as exemplified by the Sin3-HDAC complex that consists of the master scaffold protein Sin3, the Reduced Potassium Dependency 3 (RPD3)-type HDAC, and Sin3-associated structural components, such as SIN3-ASSOCIATED POLYPEPTIDE 18 (SAP18) and SAP30 (Zhang et al., 1997; Laherty et al., 1998; Wu et al., 2000; Scott and Plon, 2003; Song and Galbraith, 2006). The *Arabidopsis* genome contains six Sin3 homologs, SIN3-LIKE 1-6 (SNL1-6), four RPD3 homologs (HDA19, HDA9, HDA7, and HDA6), one SAP18 homolog, and two SAP30 homologs (SAP30 FUNCTION-RELATED 1 (AFR1) and AFR2) (Wu et al., 2000; Murfett et al., 2001; Pandey et al., 2002; Gu et al., 2013).

**FIGURE 1:**
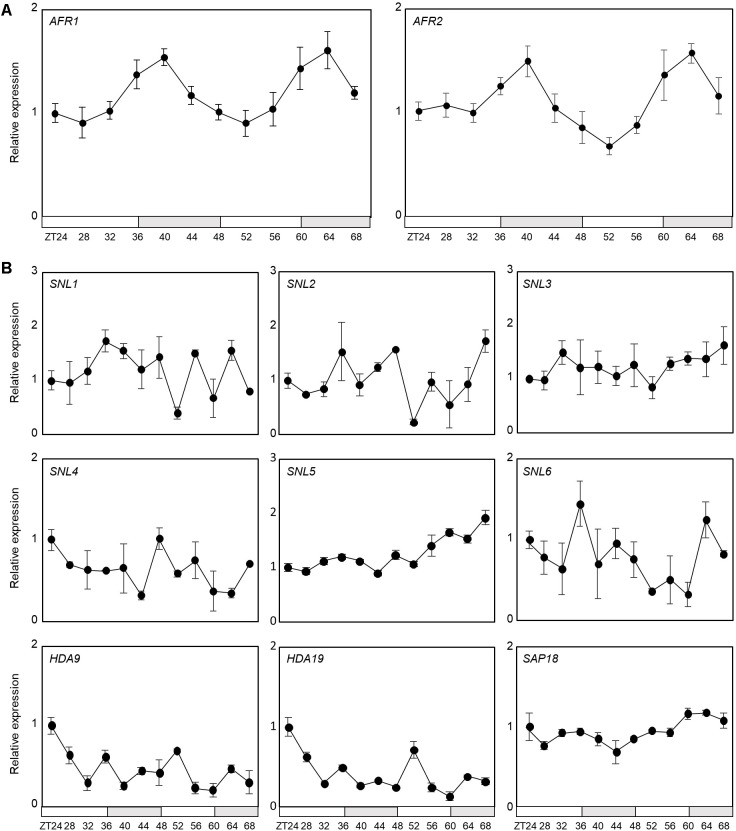
Circadian expression of *AFR1* and *AFR2*. Seedlings grown under neutral day conditions (ND, 12 h light: 12 h dark) for 2 weeks were transferred to continuous light conditions (LL) at Zeitgeber Time 0 (ZT0). Whole seedlings were harvested from ZT24 to ZT68 to analyze transcript accumulation. Transcript levels were determined by quantitative real-time RT-PCR (RT-qPCR). Gene expression values were normalized to *EUKARYOTIC TRANSLATION INITIATION FACTOR 4A1* (*eIF4A*) expression. Three independent biological replicates were averaged. Bars represent the standard error of the mean. The white and gray boxes indicate the subjective day and night, respectively. **(A)** Expression of *AFR1* and *AFR2*. **(B)** Expression of other components of Sin3-HDAC.

Notably, AFR1 and AFR2 have been identified as regulators of photoperiodic flowering, which facilitate periodic histone deacetylation at the *FT* locus (Gu et al., 2013). Considering their roles in temporal histone deacetylation, we hypothesized that the *Arabidopsis* Sin3-HDAC complex may also be implicated in circadian control. To examine the possible involvement of the HDAC complex in circadian oscillation, we first checked transcript accumulation of key components of the Sin3-HDAC complex in seedlings entrained under neutral day (ND) conditions. Quantitative real-time RT-PCR (RT-qPCR) analysis revealed that only the *AFR1* and *AFR2* genes are circadianly-regulated (Figure 1A), while the other components are not under the control of the circadian clock (Figure 1B). The *AFR* genes peaked at dusk (Figure 1A), as reported previously (Gu et al., 2013), suggesting that clock-controlled *AFR*s presumably lead to diurnal formation of the HDAC complex.

**FIGURE 2:**
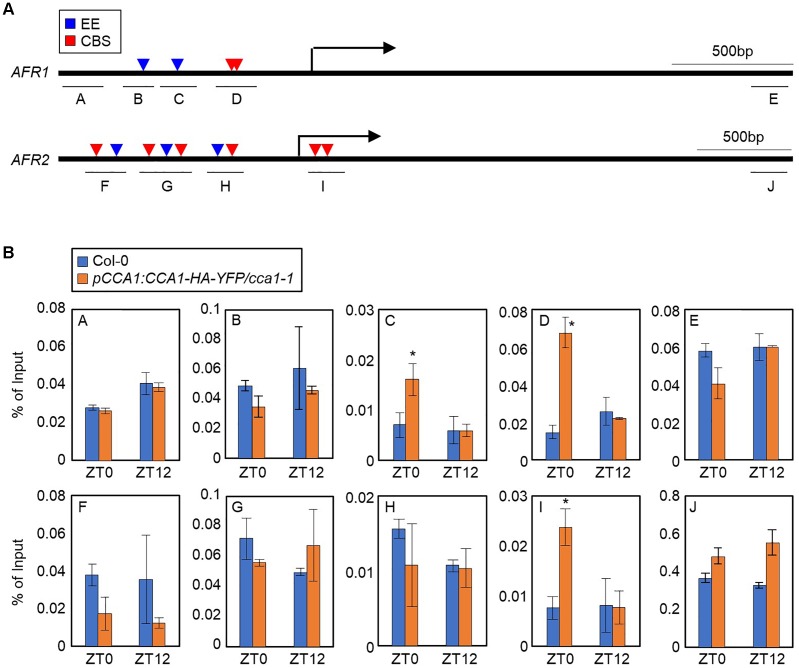
Binding of CCA1 to *AFR* promoters. **(A)** Promoter analysis of the *AFR1* and *AFR2* genes. Underbars indicate the regions amplified by PCR after chromatin immunoprecipitation (ChIP). CBS, CCA1-binding site; EE, evening element. **(B)** Binding of CCA1 to the *AFR* loci. Two-week-old plants entrained with ND cycles were subjected to LL. Plants were harvested at ZT0 and ZT12 for ChIP analysis with anti-HA antibody. Three independent biological replicates were averaged, and statistically significant differences (Student’s *t*-test, ^∗^*P* < 0.05) are indicated by asterisks. Bars indicate the standard error of the mean.

To explore the circadian component responsible for regulation of the *AFR*s, we conducted analysis of the *cis*-elements present within the *AFR* promoters. AFRs have multiple CCA1-binding sites (CBSs, AAAATCT) and evening elements (EEs, AAATATCT) in the upstream promoters (Figure 2A), which are known to be bound by CCA1 and LHY (Wang et al., 1997; Harmer et al., 2000; Michael and McClung, 2003; Nagel et al., 2015). This observation raised the possibility that CCA1 may bind to the *AFR* promoters. To examine this possibility, a chromatin immunoprecipitation (ChIP) assay was performed using plants expressing epitope-tagged CCA1 under its own native promoter (*pCCA1:CCA1-HA-YFP/cca1-1*). Total protein extracts of samples collected at Zeitgeber Time 0 (ZT0) and ZT12 were immunoprecipitated with anti-HA antibody. ChIP-qPCR analysis showed that the proximal regions of transcriptional start sites (TSSs) on the *AFR* promoters containing CBS and/or EE elements were enriched following ChIP (Figure 2B). Binding of CCA1 to the *AFR* promoter was specifically observed at dawn, but not at dusk (Figure 2B), shaping circadian expression of the *AFR*s.

**FIGURE 3:**
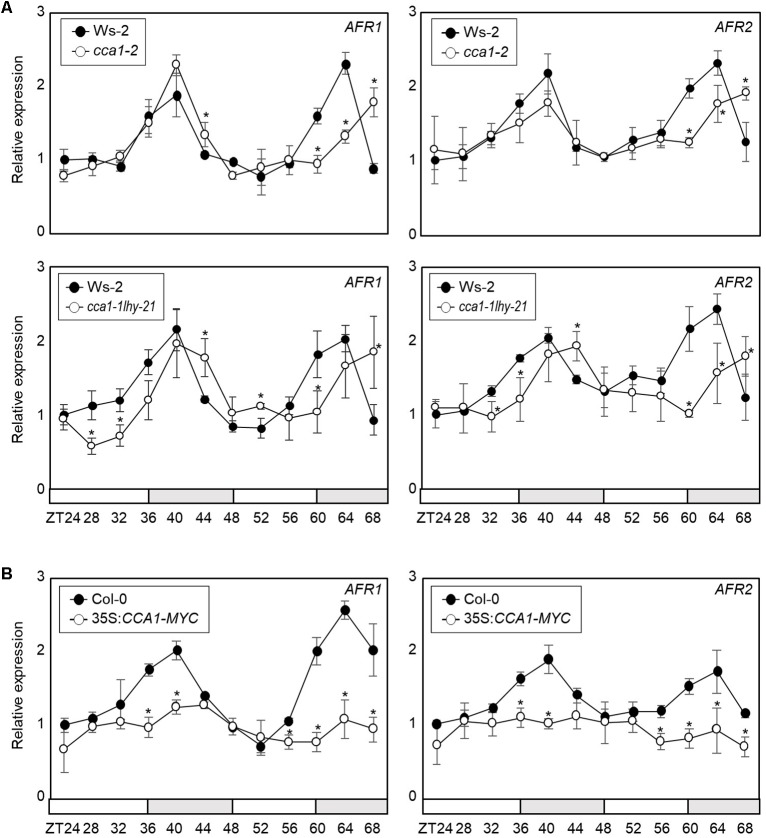
Circadian expression of *AFR*s in *CCA1*-misexpressing plants. In **(A,B)**, seedlings grown under ND conditions for 2 weeks were transferred to LL conditions at ZT0. Whole seedlings were harvested from ZT24 to ZT68 to analyze transcript accumulation. Transcript levels were determined by RT-qPCR. Gene expression values were normalized to *eIF4A* expression. Three independent biological replicates were averaged, and statistically significant differences (Student’s *t*-test, ^∗^*P* < 0.05) are indicated by asterisks. Bars represent the standard error of the mean. The white and gray boxes indicate the subjective day and night, respectively. **(A)** Expression of *AFR*s in the *cca1-2* and *cca1-1lhy-21* mutant. **(B)** Expression of *AFR*s in 35S:*CCA1-MYC* transgenic plants.

To support *AFR* regulation by the transcriptional regulator CCA1, we analyzed *AFR* expression in *cca1-2* and *cca1-1lhy-21* mutant seedlings grown under ND conditions. RT-qPCR analysis showed that the peak phase of *AFR* expression was delayed in *cca1-2* and *cca1-1lhy-21*, and higher expression of *AFR*s around the end of night was observed in the *cca1-2* and *cca1-1lhy-21* mutants compared with wild-type (Figure 3A and Supplementary Figure S1). In contrast, *AFR* expression was dramatically reduced in *CCA1*-overexpressing lines (Figure 3B). To further support the repressive role of CCA1 in *AFR* expression, we performed transient expression assays using *Arabidopsis* mesophyll protoplasts. The GUS reporter plasmids and effector plasmids harboring 35S:*CCA1-GFP* fusion were co-transfected into protoplasts (Supplementary Figure S2). Co-transfection of a reporter construct with 35S:*CCA1-GFP* resulted in lower GUS activity than the control plasmid (Supplementary Figure S2). These results indicate that CCA1 shapes *AFR* expression and enables peak expression particularly during the evening time.

### AFRs Are Involved in Circadian Oscillation

**FIGURE 4:**
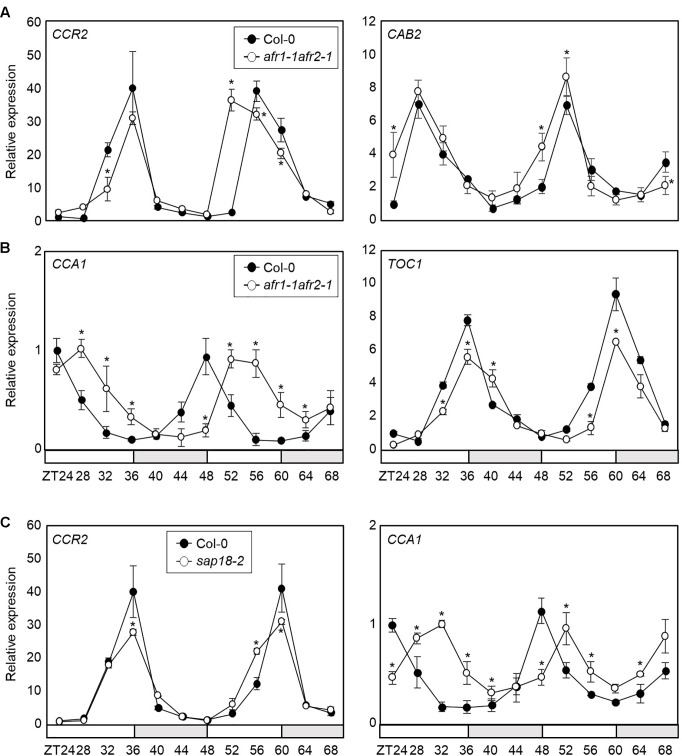
Altered circadian rhythm in the *afr1-1afr2-1* mutant. In **(A–C)**, seedlings grown under ND were transferred to LL at ZT0. Whole seedlings were harvested from ZT24 to ZT68 to analyze transcript accumulation. Gene expression values were normalized to *eIF4A* expression and represented as *n*-fold compared to the value of the wild-type sample at ZT24. Three independent biological replicates were averaged, and statistically significant differences (Student’s *t*-test, ^∗^*P* < 0.05) are indicated by asterisks. Bars indicate the standard error of the mean. The white and pale gray boxes indicate the subjective day and night, respectively. **(A)** Expression of *CCR2* and *CAB2* in *afr1-1afr2-1*. **(B)** Expression of *CCA1* and *TOC1* in *afr1-1afr2-1*. **(C)** Expression of *CCA1* and *CCR2* in *sap18-2*.

Since the AFR proteins are core Sin3-HDAC components regulated by the circadian clock, we further investigated the role of AFRs in circadian oscillation. We employed the *afr1-1afr2-1* double mutant and examined endogenous circadian behavior. RT-qPCR analysis showed that circadian output genes, *COLD CIRCADIAN RHYTHM RNA BINDING 2* (*CCR2*) and *CHLOROPHYLL A/B-BINDING PROTEIN 2* (*CAB2*), were altered in *afr1-1afr2-1* mutant seedlings compared with wild-type (Figure 4A). We also checked several core circadian oscillator genes, including *CCA1* and *TOC1*. Again, two genes were also differentially expressed in the *afr1-1afr2-1* mutant compared with wild-type (Figure 4B). In particular, the morning gene expression was delayed in *afr1-1afr2-1*. The alteration patterns of the circadian genes were dissimilar in *afr1-1afr2-1* mutant. This might be due to extensive circadian feedback network that balances 24 h clock oscillation, as observed in several previous studies (Somers et al., 2004; Ding et al., 2007; Hanano et al., 2008; Li et al., 2011).

AFRs are components of the *Arabidopsis* Sin3-HDAC complex (Gu et al., 2013). To provide further support that AFR function in circadian oscillation depends on formation of the Sin3-HDAC complex, we obtained a genetic mutant of *SAP18* and analyzed circadian oscillation. Since *SAP18* is the only member of the Sin3-HDAC components that exists as a single copy in the *Arabidopsis* genome (Zhang et al., 1997; Ahringer, 2000), we suspected that the *sap18-2* mutant could be used to reflect the roles of the *Arabidopsis* Sin3-HDAC complex. Remarkably, the *sap18-2* mutant exhibited altered circadian expression of *CCA1* and *CCR2* (Figure 4C and Supplementary Figure S3), similar to *afr1-1afr2-1*, indicating that the *Arabidopsis* Sin3-HDAC complex controls circadian oscillation.

### AFRs Bind to the *CCA1* and *PRR9* Loci and Catalyze H3 Deacetylation at Dusk

**FIGURE 5:**
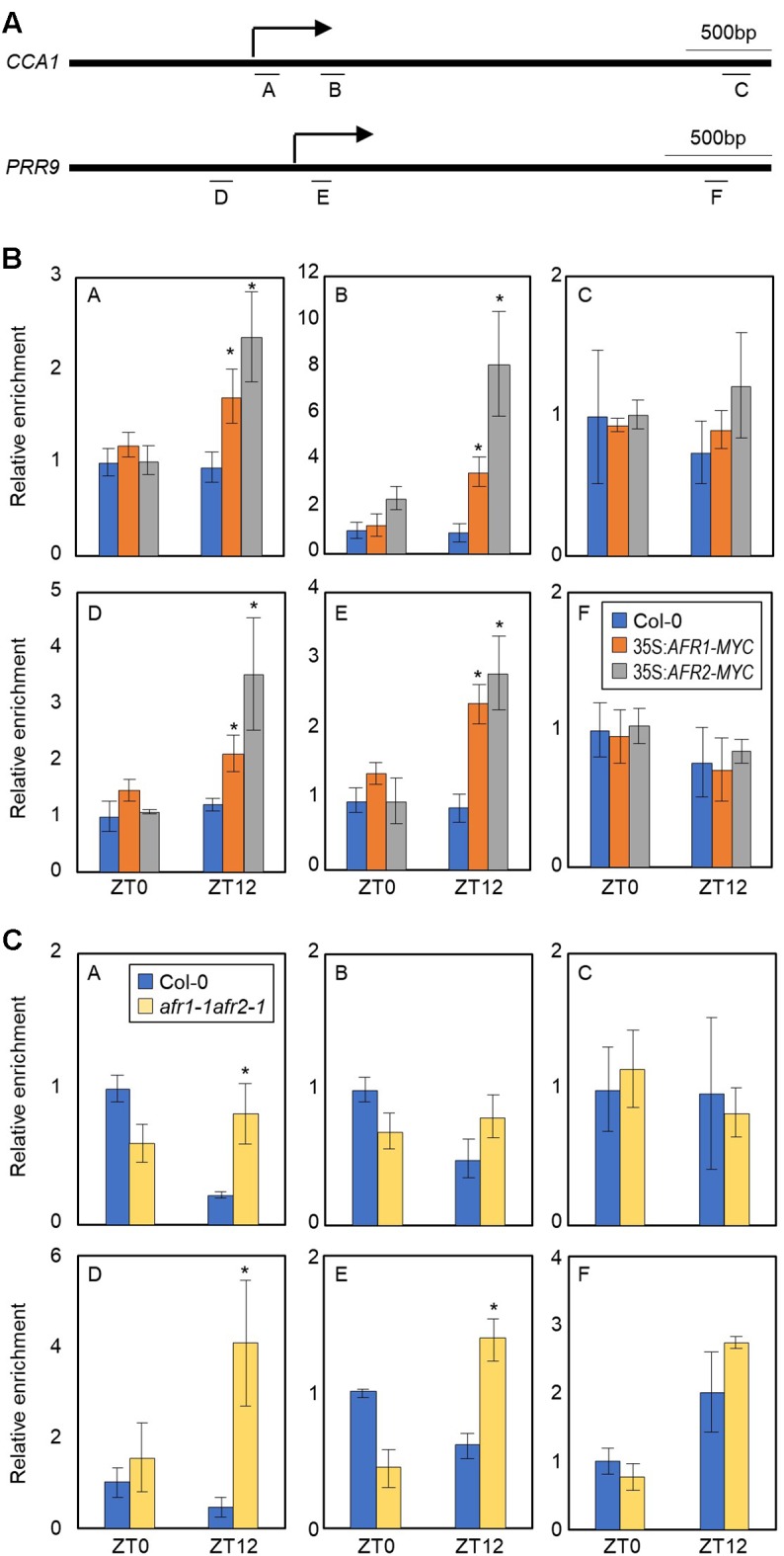
H3 deacetylation at the *CCA1* and *PRR9* loci during evening time by AFRs. In **(B,C)**, 2-week-old seedlings grown under ND were transferred to LL and harvested at ZT0 and ZT12. Enrichment of putative binding regions of AFRs in promoters of the *CCA1* and *PRR9* genes was analyzed by ChIP-PCR. Three independent biological replicates were averaged, and statistical significance of the measurements was determined by a Student’s *t*-test (^∗^*P* < 0.05). Bars indicate the standard error of the mean. **(A)** Genomic regions for ChIP analysis. Underbars represent the amplified genomic regions. **(B)** Binding of AFRs to the *CCA1* and *PRR9* loci. **(C)** Accumulation of H3ac at the *CCA1* and *PRR9* loci in the *afr1-1afr2-1* mutant. Anti-H3ac antibody was used for ChIP to assess H3ac accumulation at the loci.

AFRs most likely regulate the pace of the circadian clock possibly in association with the central oscillator(s). To identify which circadian components are regulated by the AFRs, we conducted ChIP assays using 35S:*AFR1-MYC* and 35S:*AFR2-MYC* transgenic plants. Plants were grown under ND conditions and harvested at ZT12, when AFR proteins highly accumulate (Gu et al., 2013). ChIP-qPCR analysis showed that the AFR proteins bind directly to the *CCA1* and *PRR9* loci (Figure 5A,B), while the other clock members examined were not targeted by the AFRs (Supplementary Figure S4). AFRs were primarily targeted around the TSSs of the *CCA1* and *PRR9* loci, rather than the 3′-regions of gene body (Figure 5B), which is consistent with previous observations that chromatin modification of core clock genes primarily occurs around TSSs (Hemmes et al., 2012; Malapeira et al., 2012). In addition, binding of AFRs to the *CCA1* and *PRR* loci was prominent at ZT12 (Figure 5B), when peak expression of *AFR*s was observed (Figure 1A).

The temporal recruitment of AFRs to the morning gene loci may cause periodic histone deacetylation. We examined H3 acetylation (H3ac) levels, which correlate to transcript accumulation of core clock genes (Hemmes et al., 2012; Malapeira et al., 2012), at the *CCA1* and *PRR9* promoters in wild-type and *afr1-1afr2-1* seedlings. ChIP with anti-H3ac antibody revealed that H3ac levels of the *CCA1* and *PRR9* genes were elevated at ZT0 but reduced at ZT12 in wild-type (Figure 5C), as reported previously (Hemmes et al., 2012; Malapeira et al., 2012). However, the decline of H3ac accumulation at ZT12 was impaired in the *afr1-1afr2-1* mutant (Figure 5C). Increased H3ac levels at the *CCA1* and *PRR9* loci were observed in the *afr1-1afr2-1* mutant, particularly at ZT12 (Figure 5C). These results indicate that AFRs mediate histone deacetylation at the morning gene loci to stably downregulate expression during evening time.

### The AFR Proteins Are Responsible for the Declining Phases of *CCA1* and *PRR9*

**FIGURE 6:**
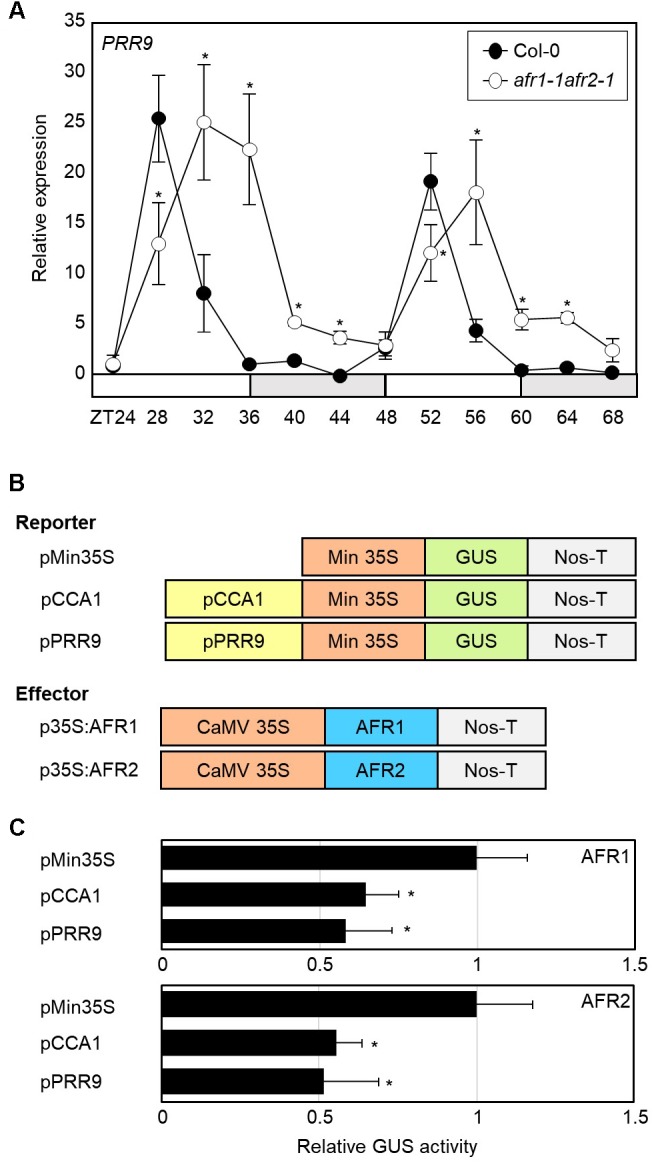
Increased expression of *PRR9* at dusk in *afr1-1afr2-1*. **(A)** Transcript accumulation of *PRR9*. Seedlings grown under ND were transferred to LL at ZT0. Whole seedlings were harvested from ZT24 to ZT68 to analyze transcript accumulation. Gene expression values were normalized to *eIF4A* expression and represented as *n*-fold compared to the value of the wild-type sample at ZT24. Three independent biological replicates were averaged, and statistical significance of the measurements was determined by a Student’s *t*-test (^∗^*P* < 0.05). Bars indicate the standard error of the mean. The white and pale gray boxes indicate the subjective day and night, respectively. **(B)** Recombinant constructs used for transient expression assays. **(C)** Transient expression analysis using *Arabidopsis* protoplasts. The core elements of *CCA1* and *PRR9* genes were inserted into the reporter plasmid. A recombinant reporter was transiently coexpressed with an effector construct containing the 35S:*AFR-MYC* construct in *Arabidopsis* protoplasts, and GUS activity was fluorimetrically determined. Luciferase gene expression was used to normalize GUS activity. Three independent measurements were averaged. Statistical significance was determined by a Student’s *t*-test (^∗^*P* < 0.05). Bars indicate the standard error of the mean.

Since the Sin3-HDAC complex catalyzes H3 deacetylation at the *CCA1* and *PRR9* loci, we speculated that circadian expression of the *CCA1* and *PRR9* genes may be shaped by diurnal H3ac accumulation. To test this possibility, we measured *CCA1* and *PRR9* expression in the *afr1-1afr2-1* mutant. In wild-type seedlings, the *CCA1* and *PRR9* genes were highly expressed in the morning, but repressed during the afternoon (Figure 4B, 6A). In contrast, decrease of *CCA1* and *PRR9* expression during afternoon was compromised in the *afr1-1afr2-1* mutant (Figure 4B, 6A). Circadian patterns of *CCA1* and *PRR9* expression were altered in the *afr1-1afr2-1* mutant background, and the increased expression of *CCA1* and *PRR9* was clearly observed at afternoon (Figure 4B, 6A).

To further support the repressive role of AFRs in *CCA1* and *PRR9* expression, we examined the extent of AFR regulation of *CCA1* and *PRR9* transcription activity in *Arabidopsis* mesophyll protoplasts. The GUS reporter plasmids and effector plasmids harboring 35S:*AFR-MYC* fusion constructs were co-transfected into mesophyll protoplasts (Figure 6B). Co-transfection of a reporter construct with 35S:*AFR1-MYC* or 35S:*AFR2-MYC* led to lower GUS activity than the control plasmid (Figure 6C). These results indicate that AFR activity limits expression of morning genes, *CCA1* and *PRR9*.

### The AFR Proteins May Interact With LNK

**FIGURE 7:**
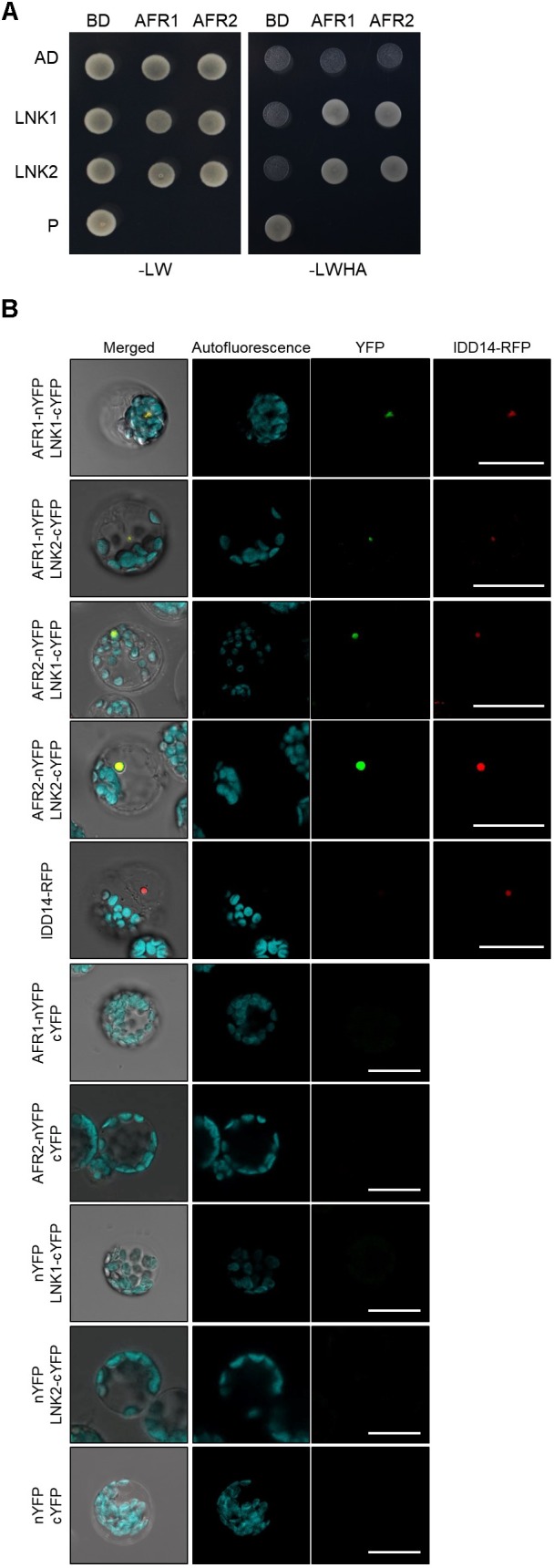
Interactions of AFRs with LNKs. **(A)** Y2H assays. Y2H assays were performed with AFR proteins fused to the DNA-binding domain (BD) of GAL4 and LNKs fused with the transcriptional activation domain (AD) of GAL4 for analysis of interactions. Interactions were examined by cell growth on selective media. -LWHA indicates Leu, Trp, His, and Ade drop-out plates. -LW indicates Leu and Trp drop-out plates. GAL4 was used as a positive control (P). **(B)** BiFC assays. Partial fragments of YFP protein were fused with AFRs and LNKs, and co-expressed in *Arabidopsis* protoplasts. The IDD14-RFP construct was used as a nuclear marker. Reconstituted fluorescence was examined by confocal microscopy. Scale bars: 20 μm.

In yeast, SAP30 is a key player in recruitment of the SAP30-Sin3-HDAC co-repressor complex to target loci (Ahringer, 2000). It is possible that the yeast SAP30 protein interacts extensively with DNA-binding transcription factors. Consistently, the *Arabidopsis* AFR1 and AFR2 proteins also frequently associate with transcription factors and guide the Sin3-HDAC complex to cognate target chromatin regions (Gu et al., 2013). To identify the molecular components that recruit the Sin3-HDAC complex to the *CCA1* and *PRR9* loci, we performed yeast-two-hybrid (Y2H) assays. Clock genes were fused in-frame to the 3′-end of the activation domain (AD) of GAL4, and each construct was coexpressed in yeast cells with a recombinant plasmid containing the GAL4 DNA binding domain (BD)-AFR fusion construct. Cell growth on selective medium showed that the transcriptional corepressors NIGHT LIGHT-INDUCIBLE AND CLOCK-REGULATED 1 (LNK1) and LNK2 specifically bind to AFR1 and AFR2 (Figure 7A and Supplementary Figure S5). The *in vivo* interactions of LNK and AFR proteins were verified by BiFC assays. Coexpression of AFR-nYFP and LNK-cYFP constructs allowed nuclear emission of YFP fluorescence, indicating physical interactions (Figure 7B). Given that the LNK corepressors act along with several DNA-binding proteins such as REVEILLE 4 (RVE4) and RVE8 (Xie et al., 2014; Perez-Garcia et al., 2015), AFRs may be recruited to the *CCA1* and *PRR9* loci at least by the DNA-binding RVE-LNK complex.

**FIGURE 8:**
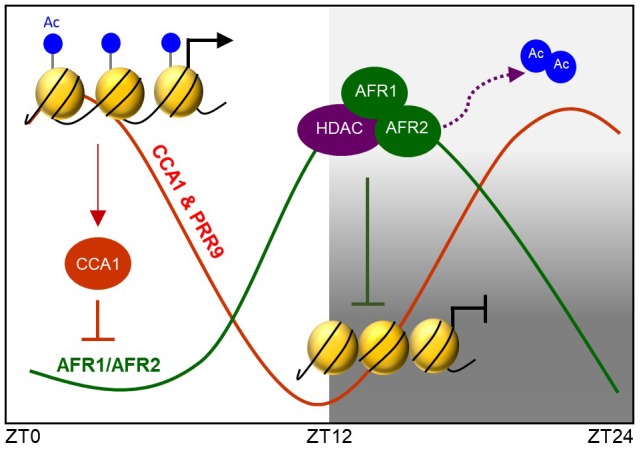
AFRs temporally regulate *CCA1* and *PRR9* genes during evening time. *Arabidopsis* Sin3-HDAC participates in regulating rhythmic expression of the *CCA1* and *PRR9* genes. The evening-expressed AFR proteins may temporally form the Sin3-HDAC corepressor complex and bind directly to the *CCA1* and *PRR9* promoters to catalyze H3 deacetylation at the cognate regions, allowing the declining phase of *CCA1* and *PRR9* expression during evening time. Binding regions of the Sin3-HDAC complex are likely specified by LNK-associated DNA-binding factors.

Taken together, the *Arabidopsis* Sin3-HDAC complex facilitates temporal H3 deacetylation at the *CCA1* and *PRR9* loci to stably regulate circadian oscillation. The AFR proteins diurnally accumulate and possibly lead to temporal association of the Sin3-HDAC complex at evening time. The AFR proteins bind specifically to the morning gene loci and facilitate H3 deacetylation at the cognate regions at dusk. Binding of the Sin3-HDAC complex to the target promoter regions is likely specified by the RVE-LNK complex (Figure 8).

## Discussion

### Chromatin Modification and the Circadian Clock

Rhythmic expression of core clock genes is intimately associated with the levels of histone modification, including H3ac and H3K4me3, at gene promoters in *Arabidopsis* (Hemmes et al., 2012; Malapeira et al., 2012). Dynamic cycles of histone modifications at the clock genes may result from transient binding of chromatin modifiers to the gene promoters. To date, several chromatin modifiers responsible for circadian control have been identified.

The SET DOMAIN GROUP 2 (SDG2)/ARABIDOPSIS TRITHORAX-RELATED 3 (ATXR3) protein is responsible for H3K4me3 deposition to activate multiple core clock genes. The H3K4me3 histone mark interferes with clock repressor binding at the core clock promoters, conferring correct timing of transcriptional repression to target clock genes (Hemmes et al., 2012; Malapeira et al., 2012). Accordingly, the *SDG2*/*ATXR3*-deficient mutants exhibit a global decrease in H3K4me3 levels and also a reduced amplitude of core clock gene expression (Berr et al., 2010; Malapeira et al., 2012; Yao et al., 2013; Pinon et al., 2017).

Circadian expression of the *CCA1* and *LHY* genes is regulated by a couple of chromatin modifiers. The *JMJ30*/*JMJD5* gene is clock-controlled and peaks at dusk (Lu et al., 2011). This pattern of *JMJ30* expression is shaped by the central oscillators CCA1 and LHY, which directly bind to the *JMJ30* promoter (Lu et al., 2011). In turn, JMJ30 promotes expression of *CCA1* and *LHY*, presumably through its histone demethylase activity (Lu et al., 2011). In addition, HDA6 and HDA19 are also implicated in the *Arabidopsis* circadian system. The HDAC proteins form a protein complex with PRRs and TPL/TPRs (Wang et al., 2013), and repress expression of *CCA1* and *LHY* by directly binding to the *CCA1* and *LHY* promoters (Wang et al., 2013). Consistently, suppression of HDAC activity leads to circadian period lengthening and compromises the transcriptional repression activities of PRR5, PRR7, and PRR9 (Wang et al., 2013).

The *Arabidopsis* Sin3-HDAC complex is a different type of HDAC complex involved in circadian oscillation. Key members of the complex, AFR1 and AFR2, are under the control of the circadian clock and form a Sin3-HDAC complex possibly in a diurnal manner to mediate periodic histone deacetylation at the *CCA1* and *PRR9* loci. AFR-dependent H3 deacetylation at the *CCA1* and *PRR9* is relevant during the evening time and thereby dampens expression specifically at dusk. Notably, even though they share the same HDAC components, the AFR-containing Sin3-HDAC complex and HDA6/HDA19-PRR-TPL complex have different binding targets in the control of circadian oscillation. Different compositions of the protein complexes may lead to different abilities in interactive protein recognition, construction of protein interaction networks and thus target chromatin binding. For instance, the AFR proteins may specifically recruit transcriptional co-regulators, such as LNKs, and facilitate new repertoires of target gene regulation in circadian control.

A significant number of HATs and HDACs participate in circadian oscillation. Specific sets of HAT and HDAC shape circadian expression of core clock genes. For instance, HAF2 adds acetyl groups specifically to the *PRR5* and *LUX* loci to facilitate the rising phase of expression (Lee and Seo, 2018), and the Sin3-HDAC complex removes the acetyl groups at the *CCA1* and *PRR9* loci to reset the acetylation state. This is likely not an exceptional case, and many biological responses are probably diurnally shaped by means of chromatin modifications (Kouzarides, 2007; Jang et al., 2011; Seo and Mas, 2014). The opposing activities of HAT and HDAC at specific genes conceivably modulate the acetylation dynamics of target chromatin regions during a day and set gene expression at the adequate level at the right time.

### Interactions of Chromatin Modifiers With DNA-Binding Transcription Factors

Histone acetyltransferases and HDACs are targeted to actively transcribed loci to control acetylation state and thereby gene expression at the genome level (Kuo and Allis, 1998; Wang et al., 2009; Peserico and Simone, 2011; Hemmes et al., 2012; Malapeira et al., 2012). However, since they have no selectivity to DNA elements, they are usually recruited to specific target loci by DNA-binding transcription factors (Todeschini et al., 2014; Bauer and Martin, 2017; Inukai et al., 2017). Interactions of chromatin modifiers with transcription factors allow elegant spatial and temporal modification of chromatin contexts (Munshi et al., 1998, 2001; Agalioti et al., 2000; Lomvardas and Thanos, 2002; Bauer and Martin, 2017).

Interactions of HDAC proteins with core clock components are crucial for refining circadian behavior in eukaryotes (Perales and Mas, 2007; Nakahata et al., 2008; Grimaldi et al., 2009). For example, in mammals, SIRT1 associates with a core transcription factor CLOCK, a positive regulator of the circadian machinery, and is recruited to the circadian gene promoters (Nakahata et al., 2008). Similarly, HDACs are associated with core clock components with DNA-binding activities in the control of circadian signaling in *Arabidopsis* (Perales and Mas, 2007). In the circadian expression of *TOC1*, the histone acetylation state seems to be regulated, at least in part, by the clock factors CCA1 and RVE8, as plants mis-expressing the MYB transcription factors exhibit an altered pattern of histone acetylation at the *TOC1* locus (Perales and Mas, 2007). CCA1 may specify repressive chromatin structures at the *TOC1* locus to regulate its expression at dawn, whereas RVE8, which has a high degree of sequence homology to CCA1, favors H3 acetylation in contrast to CCA1, most likely by antagonizing CCA1 function during the *TOC1* raising phase (Farinas and Mas, 2011). Although chromatin modifiers responsible for accumulation of H3ac at the *TOC1* locus are elusive so far, the oscillating H3ac levels are dependent on core clock transcription factors that will recruit HATs and/or HDACs to shape the waveform of *TOC1*.

AFR1 and AFR2 are recruited to the *CCA1* and *PRR9* chromatin for H3 deacetylation possibly by LNKs, although further experiments are required to prove the putative interactions. The morning-expressed LNK1 and LNK2 transcriptional coactivators lack DNA binding domains, but they interact with the *bona fide* DNA-binding proteins including CCA1, LHY, RVE4, and RVE8 to bind to core clock genes (Xie et al., 2014). Although it is unclear so far, the LNK1/2-interacting CCA1/RVEs and/or as-yet-unidentified DNA-binding proteins may transcriptionally activate *CCA1* and *PRR9* expression in the morning and also enable recruitment of the Sin3-HDAC complex to the morning gene loci to subsequently dampen expression after peak phase. The dynamic nature of histone acetylation and deacetylation depends on sophisticated interactions with transcription factors, and protein interaction networks further diversify the molecular mechanisms underlying rhythmic expression of core clock genes and thus circadian oscillation.

## Materials and Methods

### Plant Materials and Growth Conditions

*Arabidopsis thaliana* (Columbia-0 ecotype) was used for all experiments described, unless specified otherwise. Plants were grown under neutral day conditions (NDs; 12-h light/12-h dark cycles) with cool white fluorescent light (120 μmol photons m^-2^ s^-1^) at 22-23°C. The *afr1-1afr2-1* mutant was previously reported (Gu et al., 2013). *sap18-2*, *cca1-1lhy-21*, and *cca1-2* mutants were obtained from *Arabidopsis* Biological Resource Center (ABRC). The lack of gene expression in mutants was verified by means of RT-PCR.

### Quantitative Real-Time RT-PCR Analysis

Total RNA was extracted using the TRI reagent (TAKARA Bio, Singa, Japan) according to the manufacturer’s recommendations. Reverse transcription (RT) was performed using Moloney Murine Leukemia Virus (M-MLV) reverse transcriptase (Dr. Protein, Seoul, South Korea) with oligo(dT18) to synthesize first-strand cDNA from 2 μg of total RNA. Total RNA samples were pretreated with an RNAse-free DNAse. cDNAs were diluted to 100 μL with TE buffer, and 1 μL of diluted cDNA was used for PCR amplification.

Quantitative RT-PCR reactions were performed in 96-well blocks using the Step-One Plus Real-Time PCR System (Applied Biosystems). The PCR primers used are listed in Supplementary Table S1. The values for each set of primers were normalized relative to the *EUKARYOTIC TRANSLATION INITIATION FACTOR 4A1* (*eIF4A*) gene (At3g13920). All RT-qPCR reactions were performed in three independent biological replicates using total RNA samples extracted from three independent replicate samples. The comparative ΔΔC_T_ method was employed to evaluate the relative quantities of each amplified product in the samples. The threshold cycle (C_T_) was automatically determined for each reaction by the system set with default parameters. Specificity of the RT-qPCR reactions was determined by melt curve analysis of the amplified products using the standard method installed in the system.

### Yeast Two-Hybrid Assays

Yeast two-hybrid (Y2H) assays were performed using the BD Matchmaker system (Clontech, Mountain View, CA, United States). The pGADT7 vector was used for GAL4-AD fusion, and the pGBKT7 vector was used for GAL4-BD fusion. The yeast strain AH109 harboring the *LacZ* and *His* reporter genes was used. PCR products were subcloned into the pGBKT7 and pGADT7 vectors. The expression constructs were cotransformed into yeast AH109 cells and transformed cells were selected by growth on SD/-Leu/-Trp medium.

### Bimolecular Fluorescence Complementation (BiFC) Assays

The *LNK* genes were fused in-frame to the 5′ end of a gene sequence encoding the C-terminal half of EYFP in the pSATN-cEYFP-C1 vector (E3082). The *AFR* cDNA sequences were fused in-frame to the 5′ end of a gene sequence encoding the N-terminal half of EYFP in the pSATN-nEYFP-C1 vector (E3081). The IDD14-RFP construct was used as a nuclear marker (Seo et al., 2011). The expression constructs were cotransformed into *Arabidopsis* protoplasts. Expression of the fusion constructs was monitored by fluorescence microscopy using a Zeiss LSM510 confocal microscope (Carl Zeiss, Jena, Germany).

### Chromatin Immunoprecipitation (ChIP)

*pCCA1:CCA1-HA-YFP/cca1-1* and 35S:*AFR-MYC* transgenic plants were used for ChIP. Anti-MYC (06-599, Millipore), anti-HA (ab9110, Abcam), and anti-H3ac (05-724, Millipore) antibodies and salmon sperm DNA/protein A agarose beads (Millipore, Billerica, MA, United States) were used for chromatin immunoprecipitation. DNA was purified using phenol/chloroform/isoamyl alcohol and sodium acetate (pH 5.2). The level of eluted DNA fragments was quantified by quantitative real-time PCR using specific primer sets (Supplementary Table S2). The values were normalized to the input DNA level.

### Transient Expression Assays

For transient expression assays using *Arabidopsis* protoplasts, reporter and effector plasmids were constructed. The core elements of the *CCA1* and *PRR9* promoters were inserted into the reporter plasmid, which contains a minimal 35S promoter sequence and the GUS gene. To construct the p35S:AFR effector plasmids, the *AFR1* and *AFR2* cDNAs were inserted into the effector vector containing the CaMV 35S promoter. Recombinant reporter and effector plasmids were cotransformed into *Arabidopsis* protoplasts by polyethylene glycol-mediated transformation. GUS activity was measured by a fluorometric method. A CaMV 35S promoter-luciferase construct was also cotransformed as an internal control. The luciferase assay was performed using the Luciferase Assay System kit (Promega,^1^).

## Author Contributions

PJS conceived and designed the experiments. PJS wrote the manuscript with the help of HGL. HGL and CH conducted the experiments and contributed to the study design.

## Conflict of Interest Statement

The authors declare that the research was conducted in the absence of any commercial or financial relationships that could be construed as a potential conflict of interest.

## Footnotes

1 http://www.promega.com/
