# Supplementary material for: The Arabidopsis Sin3-HDAC Complex Facilitates Temporal Histone Deacetylation at the CCA1 and PRR9 Loci for Robust Circadian Oscillation
Source: Front Plant Sci. 2019 Feb 18;10:171. doi: 10.3389/fpls.2019.00171 (PMC6387943; doi:10.3389/fpls.2019.00171)
Supplement: Supplementary file 1 [file Data_Sheet_1.PDF]

Supplementary Figure

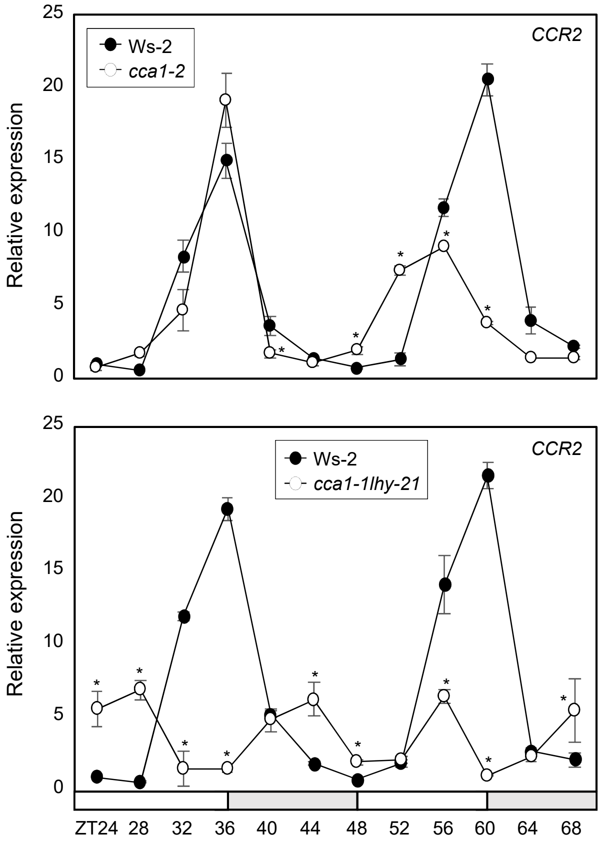

Supplementary Figure S1. Circadian expression of *CCR2* in *cca1-2* and *cca1-1lhy-21*. Seedlings grown under neutral day conditions (ND, 12h light: 12h dark) for 2 weeks were transferred to continuous light conditions (LL) at Zeitgeber Time 0 (ZT0). Whole seedlings were harvested from ZT24 to ZT68 to analyze transcript accumulation. Transcript levels were determined by quantitative real-time RT-PCR (RT-qPCR). Gene expression values were normalized to *EUKARYOTIC TRANSLATION INITIATION FACTOR 4A1* (*eIF4A*) expression. Biological triplicates were averaged, and statistically significant differences (Student's *t*-test, *P < 0.05) are indicated by asterisks. Bars represent the standard error of the mean. The white and grey boxes indicate the subjective day and night, respectively.

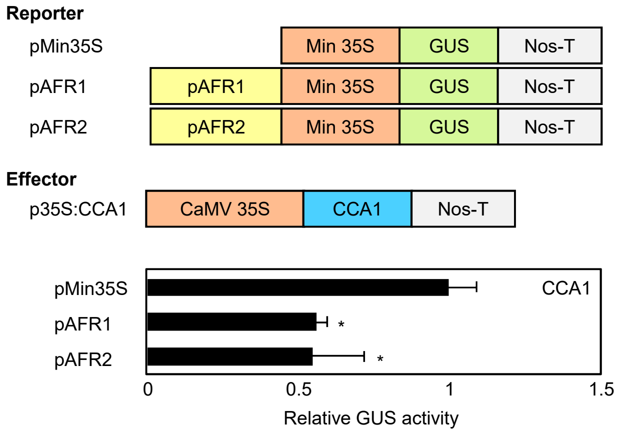

Supplementary Figure S2. Transient expression assays.
The core elements of *AFR1* and *AFR2* genes were inserted into the reporter plasmid. A recombinant reporter was transiently coexpressed with an effector construct containing the 35S:*CCA1-GFP* construct in *Arabidopsis* protoplasts, and GUS activity was fluorimetrically determined. Luciferase gene expression was used to normalize GUS activity. Three independent measurements were averaged. Statistical significance was determined by a Student's *t*-test (*$P < 0.05$). Bars indicate the standard error of the mean.

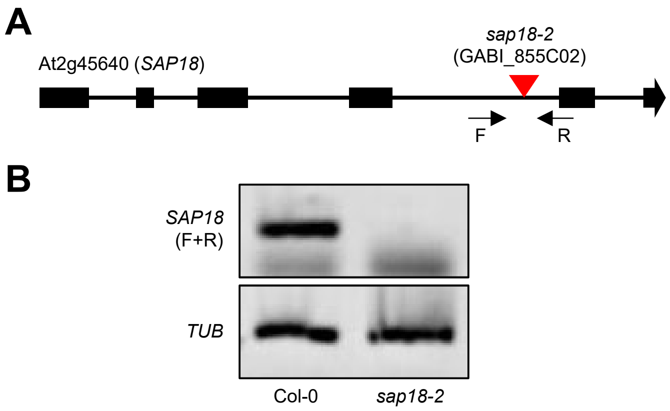

Supplementary Figure S3. Isolation of *sap18-2* mutant.

(**A**) Mapping of the T-DNA insertion site of *sap18-2* mutant. Black rectangles indicate exons. Red arrowhead indicates T-DNA insertion site.

(**B**) Transcript accumulation of *SAP18*. Two-week-old seedlings grown under NDs were harvested for total RNA isolation. Transcript accumulation was analyzed by semi-quantitative RT-PCR. The *TUBULIN BETA CHAIN 2* (*TUB*) gene (At5g62690) was used as an internal control.

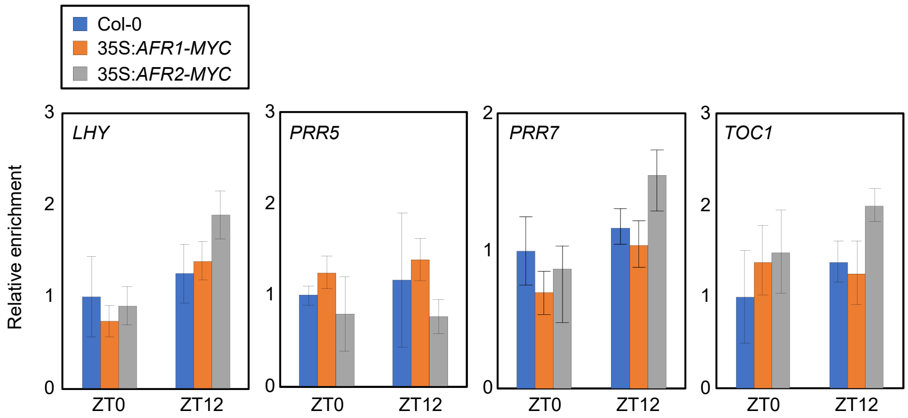

Supplementary Figure S4. Binding of AFRs to core clock gene loci.
Two-week-old seedlings grown under ND were transferred to LL and harvested at ZT0 and ZT12. Enrichment of putative binding regions of AFRs in promoters of several clock genes was analyzed by ChIP-qPCR. Biological triplicates were averaged, and statistical significance of the measurements was determined by a Student's *t*-test (*$P < 0.05$). Bars indicate the standard error of the mean.

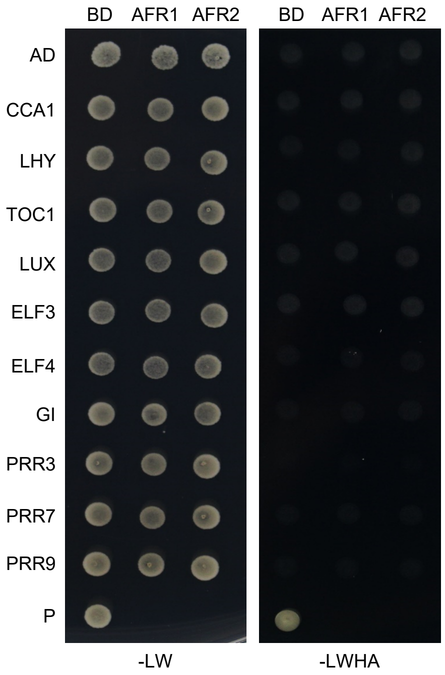

Supplementary Figure S5. Yeast-two-hybrid assays.
Y2H assays were performed with AFR proteins fused to the DNA-binding domain (BD) of GAL4 and clock components fused with the transcriptional activation domain (AD) of GAL4 for analysis of interactions. Interactions were examined by cell growth on selective media. -LWHA indicates Leu, Trp, His, and Ade drop-out plates. -LW indicates Leu and Trp drop-out plates. GAL4 was used as a positive control (P).

Supplementary Table

Primer	Usage	Sequence
ACT2-F	RT-qPCR	5′-CCATCCTCCGTCTTGACCTT
ACT2-R	RT-qPCR	5′-ACTTGCCCATCGGGTAATTC
eIF4a-F	RT-qPCR	5′-TGACCACACAGTCTCTGCAA
eIF4a-R	RT-qPCR	5′-ACCAGGGAGACTTGTTGGAC
AFR1-F	RT-qPCR	5′-CGCGGTTATCTCAAAAGGCT
AFR1-R	RT-qPCR	5′-GGCAAGCCTTCTTCATTCCT
AFR2-F	RT-qPCR	5′-CGAAAACACACAGAGGAATGG
AFR2-R	RT-qPCR	5′-TGCTCCTTTGATGGATTTGG
CCR2-F	RT-qPCR	5′-CGTTATTGATTCCAAGATCA
CCR2-R	RT-qPCR	5′-ATCCTTCATGGCTTTCTCAT
CAB2-F	RT-qPCR	5′-TTCCCAAGTAATCGAGCC
CAB2-R	RT-qPCR	5′-CCTTACCGGAGAGTTCCC
CCA1-F	RT-qPCR	5′-GATCTGGTTATTAAGACTCGGAAGCCATATAC
CCA1-R	RT-qPCR	5′-GCCTCTTTCTCTACCTTGGAGA
TOC1-F	RT-qPCR	5′-TCTTCGCAGAATCCCTGTGAT
TOC1-R	RT-qPCR	5′-GCTGCACCTAGCTTCAAGCA
PRR9-F	RT-qPCR	5′-TTGGTCCTGAGCTTGGACTTT
PRR9-R	RT-qPCR	5′-GCTTACGCTTGATGATCCGA
SNL1-F	RT-qPCR	5′-GCGAGTGTTGCACTCCTAGCT
SNL1-R	RT-qPCR	5′-TCTGCGCATGTGCTTAAAAGA
SNL2-F	RT-qPCR	5′-AGTCAAGCCCAACGGTATG
SNL2-R	RT-qPCR	5′-AGGTCAGAACGGTCAACAC
SNL3-F	RT-qPCR	5′-AACGCCGCAAGATCATCAGAG
SIN3-R	RT-qPCR	5′-ATCAGCCATACATTCAGCCTCAC
SNL4-F	RT-qPCR	5′-TTGCCAATGGGTCTCACTAAAG
SNL4-R	RT-qPCR	5′-GATTCCTAAGTTGCCTGATATTGAC
SNL5-F	RT-qPCR	5′-AGAAGAAAGCAGAAGAAAGCAACAC
SNL5-R	RT-qPCR	5′-TGAGTTAAGGCAAGGCGACAAG
SNL6-F	RT-qPCR	5′-TACCGGTGATACTAACGCGCT
SNL6-R	RT-qPCR	5′-TTGGAGTCCTGCTGCTTGAA
SAP18-F	RT-qPCR	5′-AAGCAGCGAGAAGACAAG
SAP18-R	RT-qPCR	5′-GTTCAGGTTTAGGGCGAG
HDA9-F	RT-qPCR	5′-GCCTGCATAGCAAGATGGAA
HDA9-R	RT-qPCR	5′-CCGGCGTAAAGTTGACAAAA
HDA19-F	RT-qPCR	5′-CGATATTGCCATCAACTGGG
HDA19-R	RT-qPCR	5′-AATGCCTCCTCCACTCCATC
SAP18-F	RT-qPCR	5′-AAGCAGCGAGAAGACAAG
SAP18-R	RT-qPCR	5′-GTTCAGGTTTAGGGCGAG
TUB-F	RT-PCR	5′-CTCAAGAGGTTCTCAGCAGTA
TUB-R	RT-PCR	5′-TCACCTTCTTCATCCGCAGTT
SAP18-F	RT-PCR	5′-ATCATACTAGTGAAGATTATGCTGTGAG
SAP18-R	RT-PCR	5′-TAAATTGCCACATCCAGATAATC

Supplementary Table S1. Primers used in this study.
The sizes of PCR products ranged from 80 to 300 nucleotides in length. F, forward primer; R, reverse primer.

Primer	Sequence
AFR1(A)-F	GGTGATACGTTTTAAATCATCAG
AFR1(A)-R	CGGAAAAACAGAACATATTTCC
AFR1(B)-F	GCTTAAGAATCACTCCATGAAC
AFR1(B)-R	GTTTTCGTTCCTCTCCAATG
AFR1(C)-F	CAATAGGGGTATAATCGTAACTTAC
AFR1(C)-R	GATCAAAAAAGGAAAACGAGGG
AFR1(D)-F	CCAAACGTATCCACTCCTTTC
AFR1(D)-R	GAGAGCTTTTTTACTTTTTACTCTC
AFR1(E)-F	CAGAGACACTTCATGTCTCAG
AFR1(E)-R	CTTGGCAAGCCTTCTTCATTC
AFR1(F)-F	CCTCTAGATTTCGTAGGTTTATG
AFR2(F)-R	GTCATTGTCACAGTTAACAAAGC
AFR2(G)-F	CCAAACATGTAACTTTCATATAG
AFR2(G)-R	GGATAATTGGGTATATTAGATAC
AFR2(H)-F	CTTACTAAGCAGTACTTGTTTCG
AFR2(H)-R	CCTTAGTCACGTAACTTTTTTCC
AFR2(I)-F	GATTTTCTATCAGTGTTCAAAGCTG
AFR2(I)-R	CAATACCGATAACTCTTCTTCAC
AFR2(J)-F	CCTTCACTGTGTTATGGATTTG
AFR2(J)-R	GAAGGCTTGCAAGTTTCAATC
CCA1(A)-F	CATTTCCGTAGCTTCTGGTCTCTT
CCA1(A)-R	ATCAGCTTGGATTCGATAAAGATTC
CCA1(B)-F	GAAGATGATTGTTTTAGGTGTCAAAG
CCA1(B)-R	CTGCCATGCTCTACCATAAAG
CCA1(C)-F	CAACAACAACAAGAACAAAGATATCC
CCA1(C)-R	GTATGGTTTAAAACCTGTTCTTCC
PRR9(D)-F	TCCAATTTGAATGATACATAGAGCAGCTG
PRR9(D)-R	TGGGTTTCTATTGTAATTGTGTGGCTAAGT
PRR9(E)-F	TCTCGGTAGATTAAGATCTAAAGCTCGTTG
PRR9(E)-R	CAACACTTGGTAAAACCAACAAAGCCTA
PRR9(F)-F	GAAACCAAAGGAAGAAGAAAGTG
PRR9(F)-R	TTTTTGTCAAAGCATCGATCTTTC
LHY-F	AATCTAAAGAGGTTATCACAACGGC
LHY-R	GCTGCTTCAAATCCTCTCTAACAAG
TOC1-F	TGTTAAGGGGATAAATTAGGCGAC
TOC1-R	GCTATGATACTTCCATGGCCAAA
PRR5-F	GTGGTTTGGTTTTGTGTATTGATC
PRR5-R	CATGCTCCATGATAAGTGTTAG
PRR7-F	TGGCCCGAGACAAATCTTTCTAATATCT
PRR7-R	GAGTGGAAATCGGAGACGACCATAA

Supplementary Table S2. Primers used in chromatin immunoprecipitation (ChIP) assays.
F, forward primer; R, reverse primer.
